# The dual roles of social support and coping in music performance anxiety: a systematic review of evidence from music students and professional musicians

**DOI:** 10.3389/fpsyg.2026.1904694

**Published:** 2026-07-29

**Authors:** Yuchen Wang, Yanqing Lan

**Affiliations:** 1 The Graduate School Arts & Culture, Sangmyung University, Seoul, Republic of Korea; 2 School of Music and Dance, Yichun Early Childhood Teachers College, Yichun, Jiangxi, China

**Keywords:** coping strategies, music performance anxiety, music students, professional musicians, social support, systematic review

## Abstract

**Background:**

Music performance anxiety (MPA) is prevalent among music students and professional musicians, yet the roles of social support and coping strategies remain insufficiently synthesized.

**Objective:**

This review aimed to identify the main types of social support and coping strategies involved in performance-related anxiety/stress and to examine their roles among music students and professional musicians.

**Methods:**

Following the PRISMA 2020 guidelines, a systematic search was conducted in Scopus, Web of Science, ERIC, and PsycINFO/PsycArticles. 26 empirical studies published between 1989 and 2026 met the inclusion criteria. Methodological quality was evaluated using the Mixed Methods Appraisal Tool (MMAT).

**Results:**

Five forms of social support were identified: teacher and performance-guidance support, peer and group support, family and significant-other support, professional and therapeutic support, and institutional, curricular, and career-context support. Four categories of coping strategies emerged: performance preparation and skill-based coping, somatic and psychological regulation, help-seeking and social coping, and maladaptive or risk-related coping. Overall, high-quality social support was associated with stronger psychological resources, greater performance control, and more effective help-seeking, whereas negative evaluative relationships, limited institutional support, and maladaptive coping were linked to heightened anxiety and stress.

**Conclusion:**

The regulation of MPA appears to be a dynamic and context-dependent process. Music institutions and professional organizations should move beyond reliance on individual coping by promoting curriculum-based MPA training, supportive evaluative cultures, and multi-level support systems.

## 1 Introduction

Music performance anxiety (MPA) should not be understood merely as a negative psychological reaction experienced by music students and professional musicians in stage or examination contexts. Rather, it may be defined as a multidimensional response to perceived evaluative threat in music performance situations, involving

physiological arousal, cognitive appraisal, emotional experience, and behavioral regulation (Barros et al., 2024; Herman and Clark, 2023; Kenny, 2011). Importantly, MPA is not necessarily equivalent to a pathological or deficit-based condition, but may be located on a continuum ranging from normal performance-related arousal that can support preparation and expressive engagement to severe anxiety that disrupts performance, learning, well-being, and occupational participation (Herman and Clark, 2023). When performers appraise pressure as threatening, uncontrollable, or unsupported over time, MPA may impair performance quality and learning experiences and may co-occur with avoidance, occupational stress, perfectionism, depression, and other forms of psychological distress (Fernholz et al., 2019; Pecen et al., 2018). Conversely, when performers have sufficient technical, psychological, and social resources, performance pressure may be appraised as a manageable challenge and regulated through adaptive preparation, pressure-adaptation training, and attentional control (de Bie et al., 2024; Lazarus and Folkman, 1984; Mornell and Wulf, 2019).

However, this shift from threat to challenge appraisal, as well as the technical, psychological, and social resources that performers can mobilize, cannot be separated from the specific performance and developmental contexts in which they are situated. In previous research and educational practice, MPA has sometimes been understood primarily as a problem resulting from individual anxiety proneness, insufficient technical preparation, or limited performance experience (Perdomo-Guevara, 2014). However, Perdomo-Guevara(2014) argued that MPA should not be viewed merely as an individual-level deficit, because the musical environment and performers' meaning-making of performance also shape their emotional experiences. This contextualized understanding has received further support in recent research: performance anxiety and performance-related stress among music students and professional musicians often result from the joint influence of individual characteristics, pedagogical environments, interpersonal interactions, organizational demands, teaching cultures, and available resources (Fernholz et al., 2019; Huang and Yu, 2022; Willis et al., 2024, 2026). Therefore, understanding MPA requires attention to both external social support and individual coping strategies.

Against this background, this systematic review aims to examine the major types and functions of social support and coping strategies used by music students and professional musicians when dealing with performance-related anxiety and stress. This perspective extends MPA beyond an individual psychological issue and reframes it as an educational and occupational health concern associated with training environments, social relationships, and coping resources. The findings may inform curriculum-based MPA training, support-system development in music education institutions, and long-term mental health support for professional musicians.

Accordingly, this review addresses the following research questions:

RQ1. What types of social support and coping strategies are reported in performance-related anxiety and stress among music students and professional musicians?

RQ2. What roles do social support and coping strategies play in regulating performance-related anxiety and stress among music students and professional musicians?

## 2 Theoretical framework

This review is primarily informed by the Social Support Buffering Hypothesis and further draws on the Transactional Theory of Stress and Coping to conceptualize coping processes in performance-related anxiety and stress among music students and professional musicians.

According to the Social Support Buffering Hypothesis, social support may mitigate the adverse effects of stressful events on psychological wellbeing and adaptive outcomes by providing emotional, informational, appraisal, and instrumental resources (Cohen and Wills, 1985). Within music performance contexts, teachers, peers, family members, accompanists, ensemble members, professional services, and institutional environments may constitute important sources of support because performance-related anxiety and stress commonly arise in highly evaluative social situations, such as public performances, examinations, auditions, adjudications, and professional engagements (Huang and Yu, 2022; Kenny, 2011; Tahirbegi, 2022).

At the same time, the Transactional Theory of Stress and Coping (Lazarus and Folkman, 1984) proposes that stress is not determined directly by external events but rather by individuals' appraisals of situational demands, available resources, and coping possibilities. Coping refers to the cognitive and behavioral efforts employed to manage situations that are perceived as stressful or as exceeding available resources. From this perspective, music students and professional musicians may respond to public performances, examinations, auditions, evaluations, or occupational pressures through preparation, practice, psychophysiological regulation, help-seeking, avoidance, or other coping strategies (Barros et al., 2024; Biasutti and Concina, 2014; Cupido, 2018).

This review integrates the Social Support Buffering Hypothesis and the Transactional Theory of Stress and Coping into a dynamic regulatory mechanism mediated by cognitive appraisal. Specifically, high-quality support from teachers, peers, family members, professional services, or institutions may influence performers' primary appraisal of performance situations, making music students and professional musicians more likely to interpret examinations, auditions, or concerts as “manageable challenges” rather than “uncontrollable threats.” At the same time, social support may also shape their secondary appraisal, namely whether performers believe that they can access available resources such as technical feedback, emotional reassurance, psychological or medical assistance, and institutional protection. These appraisals further influence the selection of coping strategies: when sufficient support is available, performers may be more likely to adopt adaptive strategies such as skill-based preparation, attentional and psychophysiological regulation, and help-seeking; by contrast, when support is absent, stigmatized, or inaccessible, they may turn to risk-related coping strategies such as avoidance or self-medication. Therefore, social support is not merely an external factor parallel to coping, but a contextual resource that shapes how coping strategies are selected, sustained, or constrained (Cohen and Wills, 1985; Lazarus and Folkman, 1984).

## 3 Method

This systematic review was conducted in accordance with the PRISMA 2020 Statement for literature identification, screening, and selection. The review process comprised four stages: identification, screening, eligibility assessment, and final inclusion.

### 3.1 Identification and search strategy

A comprehensive systematic search was conducted on 12 May 2026 across four major academic databases: Scopus, Web of Science (WoS), ERIC, and PsycINFO/PsycArticles. To identify relevant studies, the search strategy combined three core concepts—target population, social support/help-seeking, and anxiety/stress responses—using Boolean operators (see Table 1).

**Table 1:** Search terms and boolean logic.

Boolean logic	Core concept	Search terms
Concept 1	**Music performers/music students**	music^*^ OR musician^*^ OR “orchestra player^*^” OR “orchestral player^*^” OR conductor^*^ OR choir^*^ OR instrumentalist^*^ OR pianist^*^ OR vocalist^*^ OR cellist^*^ OR bassist^*^ OR flutist^*^ OR singer^*^ OR performer^*^ OR “conservatoire student^*^” OR “conservatory student^*^” OR “instrumental student^*^” OR “instrumental learner^*^” OR “vocal student^*^” OR “vocal learner^*^” OR “piano student^*^” OR “piano learner^*^” OR “singing student^*^” OR “singing learner^*^” OR “performance student^*^”
And Concept 2	**Social support/help-seeking**	“social support” OR “teacher support” OR “peer support” OR “family support” OR “parent support” OR “parental support” OR “institutional support” OR “mentor support” OR mentoring OR mentorship OR “emotional support” OR “informational support” OR “instrumental support” OR “appraisal support” OR “autonomy support” OR “supportive environment^*^” OR “supportive relationship^*^” OR counseling OR counseling OR “help seeking” OR “support seeking” OR “supportive feedback” OR “constructive feedback” OR “social connectedness” OR “seeking support” OR “seeking help” OR “seeking advice” OR “asking for help” OR “professional help” OR “supportive climate” OR “psychological help” OR belonging OR reassurance
and concept 3	**Anxiety/stress response**	anxiet^*^ OR stress^*^ OR distress^*^ OR “stage fright” OR “performance pressure” OR “competition pressure” OR “stage pressure”

^*^ indicates a truncation or wildcard operator used to retrieve variant word endings.

**Figure 1:**
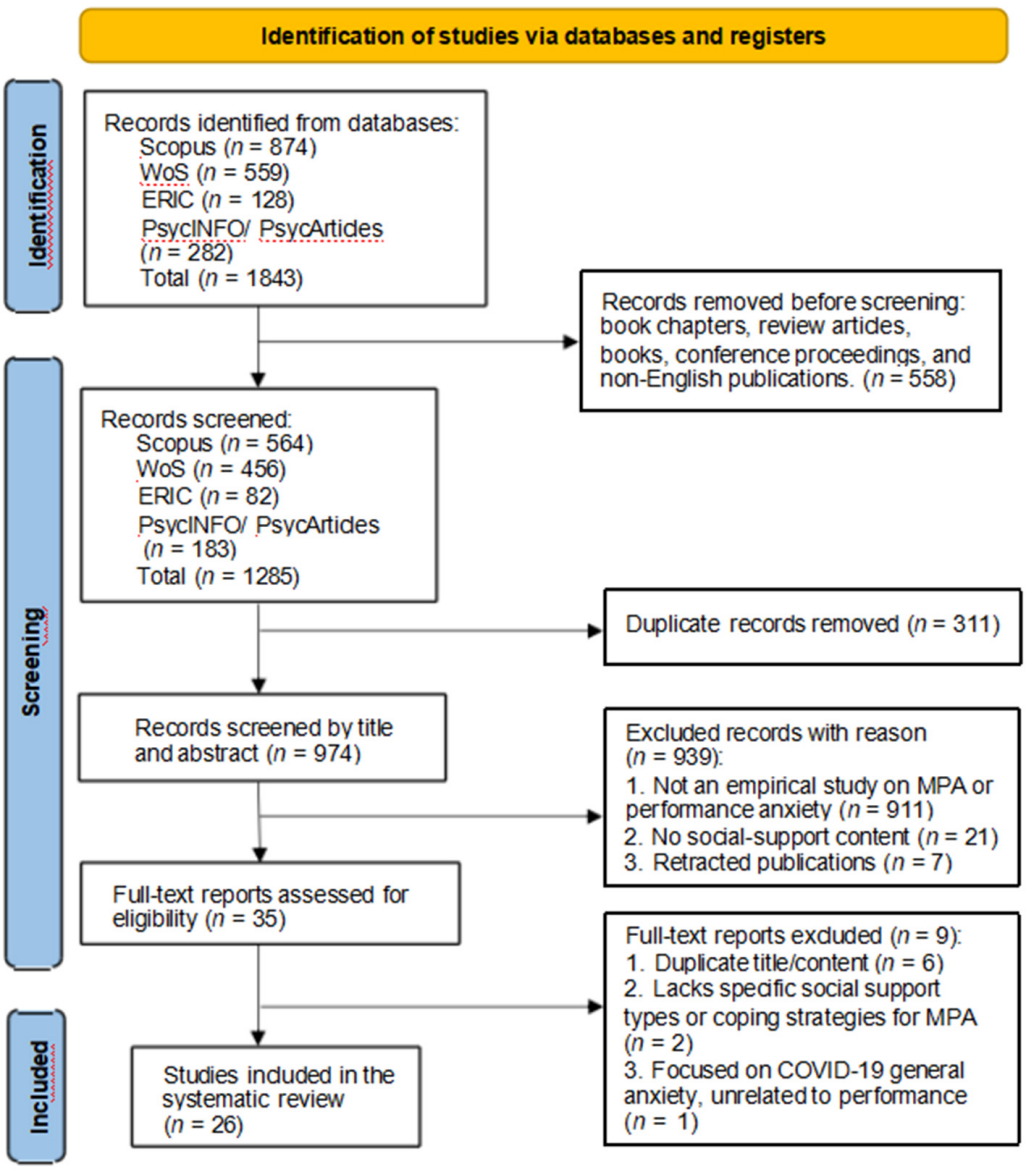
PRISMA flow diagram of study selection.

The search yielded 1,843 initial records (Figure 1). The results were subsequently restricted to English-language peer-reviewed journal articles, resulting in 1,285 records for duplicate removal and further screening.

### 3.2 Screening

Following the initial database search, 1,285 records were retrieved. After duplicate removal, 311 duplicate records were excluded, leaving 974 unique records for title and abstract screening.

The first reviewer screened the titles and abstracts according to predefined inclusion and exclusion criteria. Screening focused on whether studies were empirical investigations involving music students or professional musicians and whether they addressed MPA or performance-related anxiety and stress. Studies were further evaluated for potential relevance to the review questions by examining whether they involved social support, help-seeking behaviors, supportive environments, or coping strategies. Records that were clearly inconsistent with the aims of the review were excluded at this stage.

A total of 939 records were excluded during title and abstract screening for the following reasons:

- The study was not an empirical investigation of MPA or performance-related anxiety and stress (*n* = 911);
- The study did not address social support–related content, such as teacher support, peer support, family support, help-seeking behaviors, or supportive environments (*n* = 21);
- The article had been formally retracted (*n* = 7).

To enhance screening reliability, a second reviewer independently examined a random sample comprising 20% of the title and abstract records. Inter-rater agreement was high, with Cohen's κ ≈ 0.90. Discrepancies were resolved through discussion and re-examination of the inclusion and exclusion criteria until consensus was reached.

### 3.3 Eligibility and inclusion

Following title and abstract screening, 35 studies were retained for full-text eligibility assessment. Full texts were downloaded and examined in detail to determine whether they provided extractable empirical evidence relevant to the review questions.

The primary eligibility criteria at the full-text stage were:

- Participants were music students and/or professional musicians;
- The study addressed MPA or performance-related anxiety and stress;
- The study explicitly reported information related to social support types, help-seeking behaviors, supportive environments, coping strategies, or their roles in regulating performance-related anxiety and stress.

A total of nine studies were excluded during full-text review for the following reasons:

- Substantial duplication of study content across databases (*n* = 6);
- Insufficient reporting of specific social support types or coping strategies related to performance-related anxiety and stress (*n* = 2), including Begić (2025) and Holst et al. (2012);
- The primary focus was generalized stress, training disruption, or overall well-being during the COVID-19 pandemic rather than MPA or performance-related anxiety and stress in evaluative performance contexts (*n* = 1), such as Tamminen et al. (2026).

Disagreements during full-text review were handled using the same procedure applied during title and abstract screening. Inter-rater agreement remained high, with Cohen's κ ≈ 0.94.

Ultimately, 26 empirical studies were included in the review.

### 3.4 Data extraction and analysis

During data extraction, information was systematically collected from each included study, including the study title, participants, research design, principal findings, sources of social support, coping strategies, the roles of support and coping, and the corresponding original evidence supporting these findings. Detailed extraction results are presented in Appendix Table 1.

Following extraction, a second-order thematic coding procedure was conducted. Based on descriptions of support sources, coping behaviors, and regulatory processes reported in the studies, broader conceptual categories were developed. These categories were derived from the substantive findings reported in the original studies rather than from the exact terminology used by the authors. Original evidence excerpts were retained throughout the coding process to ensure transparency and traceability.

In this review, social support was defined as external relational, professional, or institutional resources that were either perceived or received by music students or professional musicians. Coping strategies were defined as explicitly reported behavioral actions, psychological techniques, help-seeking behaviors, or coping dimensions used to manage performance-related anxiety and stress.

Psychological resources such as self-efficacy, resilience, self-compassion, emotional intelligence, self-worth, intrinsic motivation, and self-regulated learning (SRL) were not coded as coping strategies unless they were explicitly described in the original studies as strategies or regulatory behaviors adopted by participants. Otherwise, they were interpreted as psychological resources, mechanisms, or outcomes that may be influenced by social support and coping strategies.

Methodological quality was assessed using the Mixed Methods Appraisal Tool (MMAT). In addition, each study was evaluated for its evidence contribution to RQ1, and RQ2. The MMAT assessment focused on methodological rigor across different study designs, whereas evidence contribution ratings reflected the extent to which studies directly reported sources of social support, coping strategies, and evidence regarding their roles. Neither assessment was used as a basis for excluding studies; rather, both were employed to assist in interpreting the robustness, relevance, and applicability of the evidence base.

## 4 Results

### 4.1 Methodological quality and evidence contribution to the review questions

Among the 26 included studies, 13 were rated as High quality, 11 as Moderate-to-high quality, and 2 as Moderate quality, with no studies classified as Low quality (see Appendix Table 2).

In addition to methodological quality assessed using the MMAT, this review further evaluated the extent to which each study contributed evidence to RQ1 and RQ2. For RQ1, 15 studies provided strong evidence, five provided moderate-to-strong evidence, five provided moderate evidence, and one provided low-to-moderate evidence (S15). Studies rated as providing moderate evidence were typically those that did not focus specifically on MPA or the relationship between social support and coping strategies, or those that addressed performance pressure, health resources, rituals, educational expectations, or occupational stress but did not systematically report specific sources of social support or coping strategies. The only study rated as low-to-moderate evidence for RQ1 was S15. Although this study offered rich descriptions of MPA, perfectionism, and social-evaluative pressure among singer-teachers, its evidence on social support and coping strategies was relatively dispersed and was mainly reflected in recognition needs, colleague evaluation, and experience-based self-regulation.

For RQ2, 12 studies provided strong evidence, three provided moderate-to-strong evidence, 10 provided moderate evidence, and one provided low-to-moderate evidence. Moderate evidence typically reflected indirect role evidence, cross-sectional or descriptive designs, small sample sizes, or broad research focuses. These studies suggested associations between social support or coping strategies and anxiety/stress regulation, but they did not always provide clear evidence regarding regulatory pathways or directions of effect. S15 was also rated as low-to-moderate evidence for RQ2 because it primarily reported personal experiences and social-evaluative pressure among singer-teachers, without systematically examining how social support or coping strategies regulated MPA.

By study type, the qualitative studies generally showed high methodological quality. Most qualitative studies demonstrated coherence among research questions, data collection, analysis, and interpretation. However, some cross-sectional studies provided limited reporting on sample representativeness, sampling procedures, and non-response bias. Therefore, caution is needed when interpreting causal direction and generalizability.

Overall, the MMAT assessment did not indicate systematic or serious methodological weaknesses across the included studies. However, the degree to which each study directly contributed to the review questions varied. Accordingly, this review prioritized evidence that was highly relevant to the RQs and directly reported sources of social support, coping strategies, and their roles. Studies with moderate or low-to-moderate evidence were used mainly as supplementary or contextual evidence, and their implications were interpreted cautiously.

### 4.2 Descriptive characteristics of the included studies

**Figure 2:**
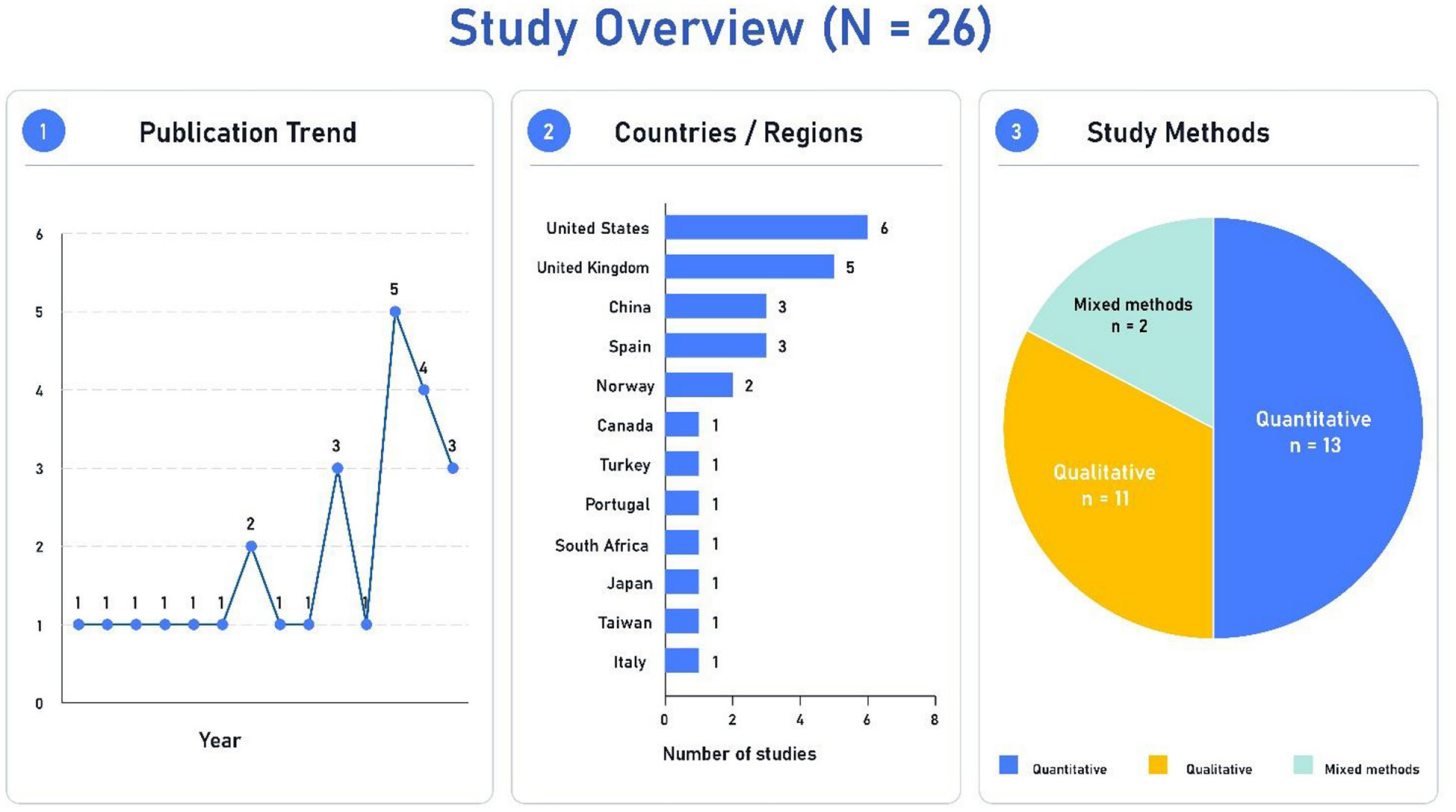
Descriptive characteristics of the included studies.

This review included 26 studies published between 1989 and 2026. Overall, early research in this area was limited, with studies appearing only sporadically between 1989 and 2020 (Figure 2). A noticeable increase in publications was observed after 2021, with 12 of the 26 included studies published between 2024 and 2026. This trend suggests growing scholarly attention to the relationships among social support, coping strategies, and MPA/performance-related stress in recent years, although the overall size of the evidence base remains limited.

In terms of geographical distribution, the included studies covered multiple countries and regions, with a general concentration in Western countries and East Asia. The United States contributed the largest number of studies (*n* = 6), followed by the United Kingdom (*n* = 5). China and Spain each contributed three studies, and Norway contributed two. The remaining studies were conducted in Canada, Turkey, Portugal, South Africa, Japan, Taiwan, and Italy, with one study from each country or region.

Regarding participants, music students were the primary population. Of the 26 included studies, 18 focused mainly on music students. The remaining studies involved professional musicians, semi-professional musicians, singer-teachers, music students and teachers, or mixed samples of students and professional musicians.

In terms of research methods, the included studies were dominated by qualitative studies and cross-sectional quantitative studies. Eleven studies used qualitative designs, making this the most common study type. These studies mainly used interviews, focus groups, life-history approaches, or interpretative phenomenological analysis to examine the experiences, support sources, and coping processes of music students and musicians in relation to performance-related anxiety and stress. Eleven studies used cross-sectional quantitative designs, including descriptive surveys, structural equation modeling, and moderation analysis. Two studies used quantitative intervention designs, and two used mixed methods. Overall, the existing evidence is mainly based on experiential descriptions and cross-sectional associations, while rigorous experimental, longitudinal, and intervention-based evidence remains relatively limited.

### 4.3 Types of social support and coping strategies in performance-related anxiety and stress

Based on the 26 included studies, five main types of social support were identified in performance-related anxiety and stress among music students and professional musicians: teacher and performance-guidance support, peer and group support, family and significant-other support, professional and therapeutic support, and institutional, curricular, and career-context support (Table 2). Coping strategies were grouped into four categories: performance preparation and skill-based coping, psychophysiological regulation, help-seeking and social coping, and maladaptive or risk-related coping (Table 3). Because a single study could contribute evidence to more than one category, the study identifiers listed in the tables overlap across categories.

**Table 2:** Classification of social support types.

Category	Core definition	Representative examples	Included studies
Teacher and performance-guidance Support	Support derived from music instruction, performance coaching, evaluative relationships, or rehearsal interactions	Major teachers, instrumental/vocal teachers, educator support, ensemble directors, accompanists, former teachers, current teachers, professors, individualized teacher discussions, teacher strategies, positive teacher attitudes	S3, S5, S6, S7, S8, S9, S13, S14, S15, S16, S18, S19, S20, S22, S26
Peer and group support	Support provided through shared experiences, peer learning, emotional understanding, group belonging, or collaborative performance experiences	Shared experiences in group music psychotherapy, peer emotional support, peer learning, friend support, ensemble-member support, group rituals, sense of community, performing with peers, music-major peers, shared musical experiences	S1, S7, S8, S9, S10, S12, S13, S17, S21, S22, S23, S25, S26
Family and significant-other support	Relational support provided by parents, family members, friends, or significant others	Parental support, family support, friend support, significant-other support, early family support, pre-university support, support from important individuals	S2, S6, S13, S16, S18, S19, S20, S21, S23, S25
Professional and therapeutic support	Formal support provided through psychological, medical, music therapy, or counseling services	Music psychotherapy, psychological counseling, psychological services, professional counseling, traditional psychotherapy, CBT-related techniques, music-enhanced therapy, vibrotactile therapeutic environments, medical/health services, professional support providers, counselors familiar with music professions	S1, S3, S4, S5, S11, S14, S23, S25
Institutional, curricular, and occupational support	Structural support provided through schools, curricula, training programs, organizations, or workplaces	MPA management courses, counseling referral systems, increased performance opportunities, examination/evaluation adjustments, non-critical training environments, health education, curriculum culture, institutional support, organizational resources, workplace resources, continuing professional development, rehearsal facilities, occupational support	S5, S10, S11, S12, S14, S17, S20, S23, S25

**Table 3:** Classification of coping strategy types.

Category	Core definition	Representative examples	Included studies
Performance preparation and skill-based coping	Managing anxiety/stress through increased task familiarity, practice quality, performance experience, and perceived performance control	Increased practice, thorough preparation, technical preparation, technical stability, simulated performances, public performance experience, mental/visual rehearsal, repertoire familiarity, acceptance of mistakes, pre-performance rituals, improvisation, performance-skills training	S5, S8, S9, S11, S14, S15, S16, S20, S22, S25
Psychophysiological regulation coping	Managing anxiety/stress through regulation of physiological arousal, attention, emotional states, and negative thoughts	Breathing exercises, grounding techniques, relaxation, visualization, meditation, yoga, physical activity, posture adjustment, positive self-talk, self-motivation, cognitive analysis, challenging negative thoughts, mindfulness/present-moment focus, self-exploratory practice	S1, S4, S5, S7, S8, S11, S13, S14, S15, S17, S22, S25
Help-seeking and social coping	Managing anxiety/stress by expressing concerns, seeking assistance, obtaining feedback, receiving understanding, or accessing professional support	Formal and informal help-seeking intentions, seeking help from teachers/professors, seeking help from medical or psychological professionals, friend support, informal support, expressing stress in groups, discussing experiences with individuals who have similar MPA experiences, feedback from trusted inner-circle members	S1, S2, S3, S4, S7, S8, S13, S15, S21, S22, S23, S24
Maladaptive or risk-related coping	Strategies that may alleviate stress in the short term but potentially maintain, exacerbate, or conceal anxiety-related problems	Avoidance, self-medication, unsupervised use of β-blockers or other medications, superstitious practices, enduring difficulties without support, excessive dependence on external validation, judgment-based practice, delayed professional help-seeking	S5, S7, S13, S14, S15, S23, S24, S25

Beyond the overall classification, the included studies also indicated contextual differences in the sources of social support and coping emphases between music students and professional musicians. Music students were mainly situated in developmental evaluation contexts, and their support was more often embedded in teaching and learning networks, including technical feedback, performance-preparation guidance, individualized dialogue, and referral-oriented support from teachers or major teachers; emotional support, peer learning, group rituals, and shared experiences from peers; and broader psychosocial resources from family members, parents, friends, and significant others (S1, S3, S5, S6, S8, S9, S16, S18, S19, S20, S21, S22, S23). By contrast, support for professional musicians was more closely linked to occupational–organizational networks and professional service systems, including colleague or orchestral support, trusted inner-circle feedback, workplace resources, organizational support, non-critical training environments, and specialized support such as psychotherapy and music-enhanced counseling (S4, S10, S11, S15).

In terms of coping strategies, music students' coping was more often organized around learning and performance preparation, such as increased practice, technical preparation, simulated performance, mental or visual rehearsal, positive self-talk, breathing and relaxation, pre-performance rituals, playing with peers, and seeking help from teachers or peers (S1, S5, S8, S9, S14, S22, S23). Professional musicians' coping more often involved professional treatment, self-management, and the use of resources within occupational contexts, including relaxation and imagery training, CBT-related techniques, music-enhanced therapy, technical stabilization, familiar repertoire, trusted inner-circle feedback, positive anxiety reappraisal, health habits, and workplace resources (S4, S10, S11, S13, S15). Therefore, the categories in Tables 1 extbfand 2 should be understood as analytical categories synthesized across studies, rather than as evidence that music students and professional musicians shared identical support environments or coped with performance-related stress in identical ways.

### 4.4 The roles of social support and coping in regulating performance-related anxiety and stress

Across the included studies, social support and coping strategies demonstrated both facilitating and constraining roles in the regulation of performance-related anxiety and stress. Although music students and professional musicians differed in their main sources of support and coping emphases, both groups appeared to reflect, at a higher level of abstraction, a support resource–cognitive appraisal–coping selection logic.

On the positive side, social support appeared to operate through three primary pathways. First, it contributed to the development of psychological resources. Social support was found to enhance or positively predict self-efficacy, emotional intelligence, resilience, self-compassion, self-worth, intrinsic motivation, pre-performance confidence, and self-regulated learning (SRL) behaviors, while reducing feelings of helplessness. These factors were in turn associated with lower levels of MPA or greater willingness to seek help (S2, S6, S16, S18, S19, S20, S21, S26). In addition, non-critical training environments and supportive groups were reported to strengthen self-esteem and emotional regulation capacities (S1, S8, S11, S15). Second, social support appeared to improve preparation quality and perceived performance control. Strategies such as increased practice, technical preparation, mental or visual rehearsal, simulated performances, repertoire familiarity, acceptance of mistakes, and pre-performance rituals were frequently associated with a stronger sense of control and reduced evaluative threat (S5, S8, S9, S11, S14, S15, S16, S20, S22, S25). In particular, collaborative support from major teachers, peers, and accompanists enabled coping strategies and relaxation skills to be integrated into everyday teaching and rehearsal practices (S22). Third, social support facilitated help-seeking and emotional expression. Participants reported seeking assistance from teachers, peers, friends, family members, healthcare professionals, counselors, and therapists to improve access to support resources (S1, S2, S3, S4, S7, S8, S13, S15, S21, S22, S23). Formal counseling, clinical music therapy, and vibrotactile therapeutic interventions also provided structured avenues through which professional musicians could transform anxiety-related experiences into issues that could be addressed through professional support (S1, S4).

At the same time, several studies highlighted factors that may limit or undermine these beneficial effects. First, negative evaluative environments and low-quality relationships may themselves become sources of stress. Negative feedback from adjudicators, low teacher empathy, abusive pedagogical practices, unsupportive organizational environments, competition, criticism, jealousy among colleagues, and excessive reliance on external validation were all reported as potential contributors to social-evaluative threat, self-doubt, and helplessness (S5, S7, S10, S12, S13, S14, S15, S21, S23, S25). Second, institutional shortcomings and insufficient occupational resources may constrain effective stress regulation. When educational institutions or workplaces lack structured MPA training programs, referral pathways, or open discussion cultures, individuals may be forced to rely primarily on limited personal resources, potentially affecting long-term wellbeing and career sustainability (S5, S10, S11, S12, S14, S17, S20, S23, S25). Third, coping strategies may also involve maladaptive or co-occurring patterns. Avoidance, superstitious practices, enduring difficulties without support, and the unsupervised use of medication (e.g., β-blockers) may provide short-term symptom relief while simultaneously maintaining anxiety, obscuring underlying psychological difficulties, or delaying access to professional support (S5, S7, S13, S14, S15, S23, S25). Notably, quantitative evidence indicated that social support seeking and support needs were positively associated with higher levels of MPA (S24). This finding suggests that help-seeking should not be interpreted solely as a protective factor but may also reflect compensatory coping efforts among individuals experiencing elevated anxiety levels.

## 5 Discussion and conclusion

### 5.1 The context-dependent and dual roles of social support in music performance anxiety

Drawing on evidence from 26 studies, this review examined the roles of social support and coping strategies in performance-related anxiety and stress among music students and professional musicians. Overall, the findings suggest that social support and coping strategies do not operate in a uniformly beneficial manner but are highly context-dependent and may exert both positive and negative influences on MPA.

High-quality social support may buffer performance-related anxiety and stress by strengthening psychological resources, enhancing perceived performance control, and facilitating effective help-seeking. Feedback, emotional understanding, preparatory guidance, and professional assistance provided by teachers, peers, accompanists, conductors, and institutional environments may increase the availability of coping resources for performers (Huang and Yu, 2022; Tahirbegi, 2022; Vengurlekar et al., 2024). Conversely, when interpersonal relationships are characterized by humiliation, excessive competition, or controlling dynamics, low-quality relationships, evaluative pressure, and inadequate institutional support may weaken the protective value of social support and increase social-evaluative threat. For example, limited teacher empathy, excessive peer competition, insufficient organizational resources, or excessive dependence on external validation may transform potentially supportive relationships into sources of anxiety and stress (Barros et al., 2024; Okan and Usta, 2021; Pecen et al., 2018). These findings are broadly consistent with the Social Support Buffering Hypothesis and the Transactional Theory of Stress and Coping. Social support does not always reduce MPA directly; rather, it may shape performers' judgments of threat, controllability, and available resources, thereby influencing their subsequent coping responses. Accordingly, MPA involves not only individual psychological regulation capacities but may also be shaped by evaluative cultures, feedback practices, teacher–student relationships, and competitive professional environments in music education and performance contexts.

Building on this point, this review further found that most studies primarily examined how social support and coping strategies reduce or buffer the negative effects of MPA, while giving less attention to the conditions under which performance-related arousal and evaluative sensitivity embedded in MPA may have functional significance. Moderate and regulable performance arousal should not be directly equated with functional impairment; it may contribute to alertness, attentional engagement, and performance readiness, and may support performance under certain conditions (Murphy et al., 2025). Findings from the included studies concerning positive anxiety reappraisal, psychological strategies, supportive training environments, and teacher, peer, and accompanist support embedded in learning and performance preparation provide indirect support for this interpretation: these factors were reported to enhance perceived performance control, flow, self-esteem, or MPA coping skills (Huang and Yu, 2022; Pecen et al., 2018; Thomson et al., 2017). Therefore, support resources and coping strategies should not be understood merely as remedial measures after MPA has already caused impairment; they should also be viewed as preconditions that influence how performance-related arousal is appraised, regulated, and used. This interpretation is consistent with recent de-pathologizing perspectives on MPA and research on pressure training (de Bie et al., 2024; Herman and Clark, 2023).

From the perspective of regulatory pathways, the role of social support in MPA is not primarily reflected in directly reducing anxiety. Several quantitative studies have shown that social support is associated with psychological resources such as self-efficacy, emotional intelligence, resilience, self-compassion, self-worth, intrinsic motivation, and self-regulated learning, and that these psychological resources are further associated with lower MPA, stronger performance confidence, or greater help-seeking intention (Huawei and Jenatabadi, 2024; Sun et al., 2026; Zarza-Alzugaray et al., 2020, 2025). From the perspectives of the Social Support Buffering Hypothesis and the Transactional Theory of Stress and Coping, social support does not simply “reduce anxiety” in a linear manner. Instead, it may first influence performers' judgments of their own competence, situational controllability, and access to help, thereby shaping whether they appraise performance pressure as a threat, a challenge, or a manageable task demand (Cohen and Wills, 1985; Lazarus and Folkman, 1984). Thus, coping in MPA contexts is better understood as a dynamic regulatory process that varies according to performance stage, stressor characteristics, and available resources, rather than as a fixed personal trait.

In addition, performers' help-seeking behaviors and their effects need to be interpreted cautiously within specific contexts. Biasutti and Concina (2014) found that “seeking social support” was positively associated with higher levels of MPA. This seemingly contradictory finding should not be interpreted simply as evidence that social support is ineffective. Rather, it may indicate that performers with higher anxiety mobilize external support more frequently when perceived control decreases, stress has already been activated, or personal resources are insufficient. Existing stress and coping research also suggests that support seeking may function both as an adaptive strategy for actively acquiring resources and as a compensatory response under high-stress conditions (Schwarzer and Knoll, 2007). Therefore, help-seeking behaviors often have preventive, adaptive, and compensatory characteristics at the same time.

Similarly, avoidance, enduring difficulties alone, superstitious practices, or medication-related coping such as the use of β-blockers should not be attributed simply to performers' irrational personal choices. The included studies suggest that medication use, avoidance, and delayed professional help-seeking are often intertwined with inadequate institutional support, evaluative pressure, barriers to psychological help-seeking, and limited access to formal services (Barros et al., 2024; Okan and Usta, 2021; Pecen et al., 2018). For example, some studies reported that students or performers used medication, β-blockers, or self-medication to manage MPA-related physiological symptoms. However, such strategies may provide only short-term and private symptom relief, without addressing the cognitive, pedagogical, or institutional factors underlying anxiety (Barros et al., 2024; Casanova et al., 2025; Okan and Usta, 2021). More importantly, formal help-seeking in music education and professional performance contexts is often constrained by stigma, limited service accessibility, and insufficient professional fit, making performers more likely to rely on informal support or hidden coping strategies (Dews and Williams, 1989; Pecen et al., 2018; Williamon and Thompson, 2006). Therefore, self-medication may appear to provide immediate and private symptom control, but unsupervised medication-related coping may also obscure deeper psychological, pedagogical, or institutional problems, delay professional intervention, and reinforce a culture in which MPA is expected to be hidden.

Overall, the findings suggest a dynamic process in which support quality is associated with psychological resources, which may shape situation appraisal and subsequent coping responses. Future research should move beyond cross-sectional associations and employ longitudinal, intervention-based, and process-oriented designs to further examine how different sources of social support influence the development of MPA through psychological resources and coping strategies.

### 5.2 Implications for music education and support system development

The findings of this review have several implications for educational interventions and support-system development related to performance-related anxiety and stress in music contexts.

First, support for MPA should not be limited to short-term anxiety reduction immediately before performances but should be embedded earlier within music learning and training processes. Evidence from the included studies suggests that early support from family members and teachers may be associated with performance self-efficacy, pre-performance confidence, and performance quality. Accordingly, MPA support may benefit from being introduced during pre-tertiary music training, with greater attention given to how parents and early teachers provide stable, encouraging, and non-shaming feedback environments. In addition, support priorities may differ across developmental and professional stages. Younger learners may benefit primarily from confidence building and psychologically safe feedback, higher education students may require curriculum-based MPA training and peer support, whereas professional musicians may benefit more from organizational resources, occupational health services, and workplace support.

Second, music institutions may consider integrating MPA management into regular curricula and performance training. Although students often use strategies such as breathing exercises, relaxation techniques, mental rehearsal, physical activity, simulated performance, positive self-talk, and task-oriented musical and technical self-instruction, these approaches may remain fragmented without structured guidance. Therefore, MPA education should move beyond general psychological regulation and give greater attention to musical and technical self-instruction linked to specific performance tasks. Such instruction is usually developed in lessons, consolidated through individual practice, and used during performance to regulate attentional focus, posture, movement, breathing, timing, and technical execution. Previous research suggests that an external focus of attention can support musical expression and technical precision (Mornell and Wulf, 2019), while music-physiological and motor-learning-oriented teacher training may support posture, movement organization, and bodily awareness (Hildebrandt and Nübling, 2004). Stage disposition may also serve as an integrative performance-training concept connecting psychological, physiological, and technical preparation (Hildebrandt, 2009). Accordingly, embedding performance preparation, psychophysiological regulation, acceptance of mistakes, cognitive reappraisal, attentional focus, music-physiological awareness, stage disposition, and help-seeking skills into coursework, workshops, and rehearsals may help position MPA management as an ongoing part of musical development rather than a crisis-driven response. This curriculum-based approach is also consistent with recent meta-analytic evidence showing that structured mindfulness- and acceptance-based interventions may reduce MPA and support adaptive psychological functioning, although their mechanisms require further verification (Zhang et al., 2026).

Third, clearer support pathways may be needed among teachers, peers, and professional services. Teachers, friends, and peers often serve as the most accessible sources of informal support but cannot replace psychological or medical services. Consequently, music educators may benefit from developing basic competencies in identifying MPA-related difficulties, providing supportive feedback, and facilitating referrals when necessary. Peer-support groups, shared performance experiences, and non-evaluative discussion spaces may also function as accessible entry points to professional support and reduce reliance on ineffective self-management strategies.

Fourth, music institutions and professional organizations may need to pay greater attention to the influence of evaluative culture on MPA. The findings suggest that negative adjudication experiences, excessive competition, limited teacher empathy, insufficient institutional support, and unsupportive occupational environments may contribute to performance-related anxiety and stress. Accordingly, intervention efforts may extend beyond individual relaxation techniques to include developmental feedback practices, low-risk performance opportunities, process-oriented assessment approaches, and organizational cultures that encourage open discussion of MPA.

Finally, the available evidence suggests that the effects of social support may vary across student groups. For example, social support appeared more strongly associated with self-worth and intrinsic motivation among female vocal students, whereas teacher and peer support showed stronger associations with self-efficacy among male students. In addition, future support design should not overlook instrument-related and bodily dimensions of MPA. Evidence from classical music students suggests that pre-performance anxiety, catastrophizing, and bodily complaints may vary by age, gender, and instrument group and may be associated with self-rated changes in performance quality (Sokoli et al., 2022). Although these findings should be interpreted cautiously because they are primarily based on cross-sectional evidence, they suggest that future support initiatives may benefit from considering individual differences in the development of psychological resources rather than relying on uniform support approaches.

### 5.3 Limitations

Several limitations should be acknowledged. First, this review included only studies published in English. As a result, relevant evidence published in Chinese, German, French, Spanish, or other languages may have been excluded. Given that music education systems and performance cultures vary considerably across countries and regions, this language restriction may have limited the representation of social support and coping processes in diverse cultural contexts.

Second, the review focused primarily on peer-reviewed publications. Although multiple databases were searched systematically, unpublished studies, doctoral dissertations, conference proceedings, and institutional reports may not have been captured. Considering that MPA interventions, psychological support programs, and occupational health initiatives are sometimes implemented as institutional or clinical projects, potentially relevant evidence may remain outside the formal publication system.

Third, substantial heterogeneity existed across the included studies in terms of participant characteristics, measurement approaches, research settings, and methodological designs. Some studies focused on music students, whereas others examined professional musicians. Likewise, some studies measured MPA directly, while others investigated performance-related stress, occupational stress, or help-seeking intentions. Although such diversity contributes to a broader understanding of the topic, it also limits direct comparisons across studies.

Finally, while the overall methodological quality of the included studies was acceptable, several studies reported limitations related to sampling procedures, measurement reporting, mixed-methods integration, or intervention descriptions. In addition, some studies contributed only indirect evidence to the review questions. Consequently, individual findings should be interpreted cautiously, and results from single studies should not be generalized to all music students or professional musicians.

### 5.4 Future research

**Figure 3:**
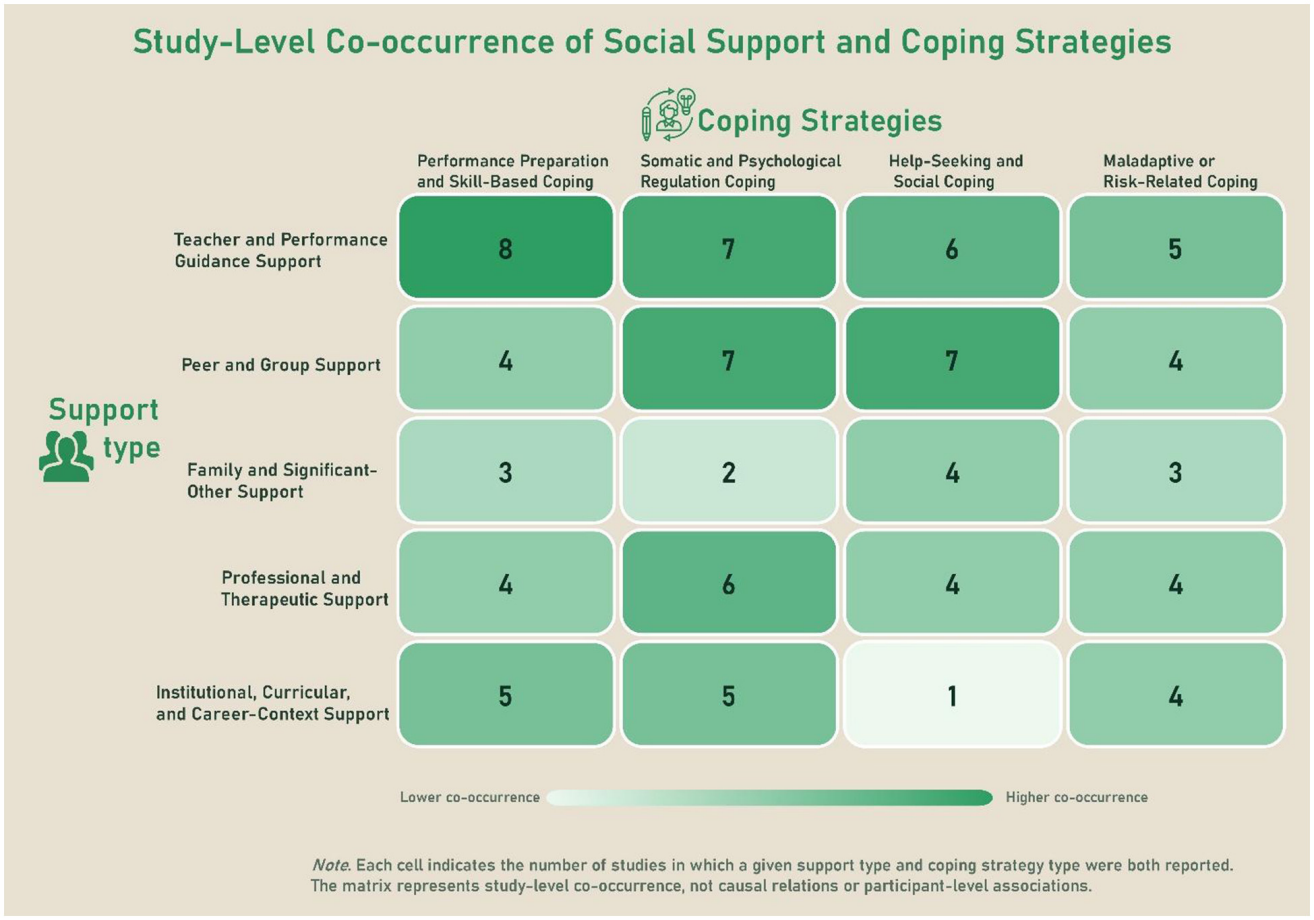
Study-level co-occurrence matrix of social support types and coping strategy types.

Based on the coding of social support types and coping strategy types across the 26 included studies, a study-level co-occurrence matrix was constructed to identify patterns and gaps in the existing evidence (Figure 3). Each cell in the figure indicates the number of studies in which a given type of social support and a given type of coping strategy were reported together. Therefore, the matrix reflects study-level co-occurrence and the distribution of existing research attention, rather than direct evidence of causal relationships, participant-level statistical associations, or the relative strength of underlying mechanisms. Higher co-occurrence may partly reflect methodological path dependency; that is, existing research instruments may be more likely to capture teacher support, practice preparation, and skill-based coping, while less readily capturing institutional support, occupational resources, or informal help-seeking.

The highest co-occurrence was observed between teacher and performance-guidance support and performance preparation and skill-based coping, with this combination appearing in 8 studies. This pattern indicates that existing MPA research has most frequently documented the intersection between teacher support and skill-based coping, but it may also reflect the relative visibility and measurability of these constructs in current research designs. Relatively high co-occurrence was also found between teacher support and psychophysiological regulation coping, and between peer and group support and psychophysiological regulation coping, each appearing in seven studies. This indicates that current research has also paid considerable attention to how teachers and peers support relaxation, breathing, mental rehearsal, emotional regulation, and body-based coping.

By contrast, only one study reported the co-occurrence between institutional, curricular, and career-context support and help-seeking and social coping. This is a notable gap because institutions, curricula, professional organizations, and workplaces are precisely the settings in which formal referral systems, counseling access, MPA management courses, peer-support programs, and occupational health services can be developed. Future research should therefore examine whether institutional support mechanisms can increase help-seeking intentions, reduce stigma, and improve long-term stress regulation.

Another underexplored area concerns the relationship between family and significant-other support and psychophysiological regulation coping, which appeared in only two studies. This suggests that family support has often been treated as a general emotional or background resource rather than as part of concrete coping processes. Future studies could examine how parents, significant others, and early teachers shape musicians' understanding of mistakes, evaluation, bodily tension, and performance pressure, and whether such early support influences later regulation strategies.

Overall, the matrix suggests that future research should move beyond simply identifying support sources and instead examine how specific forms of social support contribute to the development, selection, and sustained use of coping strategies. Given that the current evidence base remains dominated by qualitative accounts and cross-sectional associations, longitudinal, intervention-based, and process-tracing designs are needed to clarify whether and how different support types are transformed into stable MPA coping capacities during practice, rehearsal, performance, and help-seeking situations.

## Data Availability

The original contributions presented in the study are included in the article/Supplementary material, further inquiries can be directed to the corresponding author.
