# Supplementary material for: The dual roles of social support and coping in music performance anxiety: a systematic review of evidence from music students and professional musicians
Source: Front Psychol. 2026 Jul 29;17:1904694. doi: 10.3389/fpsyg.2026.1904694 (PMC13462238; doi:10.3389/fpsyg.2026.1904694)
Supplement: Supplementary file 1 [file Supplementary_File_1.docx]

Appendix Material

**Table 1. Characteristics, Evidence Extraction, and Coding of Included Studies**

| No. | Study | Participants | Research Design (MMAT) | Social Support Sources | Supporting Evidence | Coping Strategies | Supporting Evidence | Role of Support/Coping | Supporting Evidence |
| --- | --- | --- | --- | --- | --- | --- | --- | --- | --- |
| S1 | An explanatory sequential pilot inquiry on music therapy and performance anxiety in university music education majors | Music students | Mixed methods | Peer group; supportive therapeutic environment; music therapist | “The group context of the sessions and interacting with others with shared experiences of MPA served as a helpful resource in combating MPA.” “Talking to the other participants was also really helpful... people who understood...” | Box breathing; grounding; guided visualization/meditation; improvisation; relaxation; challenging negative thoughts | “Participants ... considered box breathing, grounding and visualization exercises/meditation and improvisation the most effective therapy strategies...” “...breathing/relaxation exercises... to discuss my own negative thoughts by acknowledging them and challenging them.” | Supportive group context and specific techniques helped short-term symptom management. | “Participants ... mood and anxiety ... revealed statistically significant changes...” “Anxiety scores decreased and mood scores increased...” |
| S2 | Associations between social support and help-seeking intention regarding music performance anxiety among Chinese music students: mediation via resilience and self-compassion | Music students | Quantitative descriptive / SEM | General perceived social support | “social support, resilience, self-compassion, FHSI, and IHSI were positively correlated with each other...” | Formal help-seeking intention; informal help-seeking intention | “FHSI” and “IHSI” were included as outcome variables in the mediation model. | Social support promoted help-seeking intention through resilience and self-compassion. | “Social support was not directly related to formal help-seeking intention, but its indirect effects through resilience and self-compassion were significant... For informal help-seeking, social support had both direct and indirect effects...” |
| S3 | Awareness and incidence of health problems among conservatoire students | Music students | Quantitative descriptive | Instrumental teachers; medical/professional services | “Students showed a significant inclination to go first to their instrumental teacher for advice about health and psychological problems, before appropriate medical practitioners.” | Seeking advice from teachers; seeking professional help | “...broadening their awareness of the health issues associated with performing professionally and of the places where they could seek professional help.” | Teachers were accessible first-line support, but professional referral was needed. | “go first to their instrumental teacher... before appropriate medical practitioners.” |
| S4 | Clinical Trial of a Music Generated Vibrotactile Therapeutic Environment for Musicians: Main Effects and Outcome Differences Between Therapy Subgroups | Professional musicians | Randomized controlled trial | Psychotherapeutic counselling; music-enhanced counselling; vibrotactile therapeutic environment | “Fifty-four professional symphony orchestra musicians... randomly assigned to... traditional psychotherapeutic counseling, counseling supplemented with music, or counseling supplemented with music plus vibrotactile sensations.” | CBT-based techniques; relaxation; imagery; music-enhanced therapy | “Treatment conditions were based on cognitive-behavioral techniques including relaxation training and imagery.” | Structured therapeutic support offered a feasible route for MPA management. | “music-enhanced therapies were just as effective as traditional counseling.” |
| S5 | Conservatory Students’ Music Performance Anxiety and Educational Expectations: A Qualitative Study | Music students | Qualitative | MPA-management course; counselling/psychological support; more stage experience; exam-format adjustment | “Course for MPA management in the curriculum (9).” “Psychologist’s support, counseling (4).” | Positive self-talk; self-motivation; posture adjustment; imagination; playing with peers; breathing; medication; meditation; superstition | “Positive self-talk (20), Self-motivation (6), Changing posture... (5), Imagination (5), Playing with peers (3), Breath exercises (3), Medication (2), Meditation (2), Superstitious beliefs (2).” | Personal strategies were often unsystematic; institutional support was needed; negative jury evaluation intensified MPA. | “participants ... used physical and cognitive coping strategies... but positive self-talk was the most used.” “Participants want detailed and theoretical knowledge about coping with MPA...” |
| S6 | Effects of social support on music performance anxiety among university music students: chain mediation of emotional intelligence and self-efficacy | Music students | Quantitative descriptive / SEM | Parental support; teacher support; peer support | “all three forms of parental, teacher, and peer support were positively associated to both self-efficacy and emotional intelligence.” | None reported | — | Social support affected MPA through emotional intelligence and self-efficacy. | “self-efficacy and emotional intelligence emerged as significant mediators of the relationship between social support and music performance anxiety.” |
| S7 | Emotional Competence Within the Stress Coping Strategies of Music Education Students | Music students | Qualitative | Professors/teachers | “The participants indicated that they reached out to professors for support ... however, they felt as though very few music professors were sympathetic toward the stressors the students were experiencing...” | Stress awareness; “grin and bear it”; self-care; seeking support | “awareness of stress, ‘grin and bear it,’ traditional self-care, and support.” | Teacher support could help, but limited empathy weakened support; emotional competence needed development. | “Minimal evidence was found for half of the skills and for some students they were nonexistent, thus indicating a need for growth in emotional competency.” |
| S8 | Higher music education students’ experiences and management of performance anxiety: A qualitative study | Music students | Qualitative | Teachers; peers; institutional support | “Teachers and peers were identified as integral to sustaining students’ well-being and MPA regulation efforts. A positive teacher attitude toward the management of MPA appeared to encourage students’ help-seeking efforts.” | Increased practice; mental skills; preparation; help-seeking; self-regulation | “The most common MPA coping strategies mentioned were increasing individual practice time, and mental skill practices.” “All students mentioned the importance of preparation...” | Preparation enhanced confidence and self-efficacy; teacher and peer support sustained regulation efforts. | “preparation... was associated with self-confidence, leading to higher self-efficacy beliefs, which in return had a positive impact on their management of MPA.” |
| S9 | How music education students embrace pre-performance rituals to enhance their craft | Music students | Mixed methods | Educators; peers; ensemble group | “critical role of educators and peers in shaping these practices.” “Group rituals... strengthen feelings of camaraderie and support among ensemble members.” | Individual rituals; group rituals; symbolic actions; direct performance strategies | “these practices range from symbolic actions to direct performance strategies.” “93% of participants incorporating rituals into their preparation.” | Rituals supported preparation, belonging, and group cohesion; direct MPA reduction was not tested. | “significant role in enhancing individual and group performance and in fostering community and belonging.” |
| S10 | “I feel like a fish out of water”: interpreting the occupational stress and well-being experiences of professional classical musicians | Professional musicians | Qualitative | Colleagues/orchestral musicians; organizational resources; workplace support | “emotional support from the orchestral musicians...” “Participants often relied on personal resources as opposed to available workplace resources.” | Preparation; personal resources; stress appraisal; problem-solving | “Participants’ appraisals were informed by... self and other comparison, and preparation.” “relied on personal resources...” | Social support could buffer occupational stress, but insufficient workplace support individualized coping. | “organizational interventions targeted at continuing professional development and social support are appropriate to help musicians cope more effectively with demands.” |
| S11 | Intensive opera training program effects: A psychological investigation | Professional/semi-professional musicians | Quantitative non-randomized | Psychologically supportive, non-critical training environment; teaching team | “Curriculum was taught in an integrative and psychologically supportive environment where singers worked musically, dramatically, and physically without fear of criticism.” | Vocal coaching; performance technique; visualization; yoga; movement; improvisation; career planning | “OperaWorks’ intensive... programs offered classes in performance technique, acting, improvisation, coaching, Alexander Technique, yoga, conducting, physical theater, audition preparation, career planning, and marketing.” | Supportive multi-modal training improved psychological resources and reduced anxiety-related risks. | “increases in global flow... self-esteem... Decreases... anxiety... shame.” |
| S12 | “It made me feel helpless because I couldn’t control anything”: Occupational stress and well-being experiences of conservatoire music students | Music students | Qualitative | Organizational social support; curriculum/cultural support; interpersonal resources | “Participants highlighted the personal resources, psychological skills and problem-solving, and the organisational resource of social support to manage stressful experiences.” | Personal resources; psychological skills; problem-solving; social support | “Participants commonly appraised demands as a threat...” “personal resources, psychological skills and problem-solving.” | Social support and psychological skills helped manage stress, but threat appraisal and lack of control limited regulation. | “Findings suggest interventions targeting the conservatoire culture and curriculum need exploring to create a positive learning culture and support students to cope with demands.” |
| S13 | “It’s Your Problem. Deal with It.” Performers’ Experiences of Psychological Challenges in Music | Musicians/performers | Qualitative | Friends; family; self-help literature; teachers; professional help | “Primary sources of support included friends, family and self-help literature. Professional help was predominantly sought for physical problems. The impact of teachers was paramount...” | Healthy habits; positive anxiety reappraisal; psychological strategies; self-management; communication/organizational skills | “elite performers... reported positive health habits, philosophical views of performance, health and life, positive anxiety reappraisal, and use of various psychological strategies...” | Support could facilitate development, while abusive teachers, unsupportive environments, comparison, and competition increased psychological challenges. | “The most negative aspects recounted included abusive teachers, unsupportive environments, social comparison, competition, and disillusionment after entering the profession.” |
| S14 | Music performance anxiety in Portuguese higher education: Contextual factors, perceptions, and strategies | Music students and teachers | Qualitative | Teachers; institutional support; teacher–student–psychologist collaboration | “teachers’ strategies to counter MPA are limited.” “lack of institutional support all contribute to the high levels of MPA.” “need for MPA to be discussed... with teachers, students, and psychologists.” | Technical/musical preparation; breathing; physical exercises; mental rehearsal; cognitive understanding; relativizing mistakes; medication/beta-blockers | “breathing and physical exercises...” “mental rehearsal...” “Relativise mistakes...” “Medication... Inderal... beta-blockers.” | Teacher strategies helped but were limited; institutional culture and lack of support maintained high MPA; medication carried risks. | “institutional culture, style of teaching, and lack of institutional support converge to maintain high levels of MPA...” “medication... without medical advice...” |
| S15 | Music Performance Anxiety, Perfectionism and Its Manifestation in the Lived Experiences of Singer-Teachers | Professional musicians/teachers | Qualitative | Supportive colleagues; trusted inner circle; teachers/mentors; student/colleague recognition | “I am lucky in my current institution, I have very supportive colleagues.” “Having a trusted inner circle was an important coping mechanism for them.” | Technical stabilization; familiar repertoire; lessons with own teacher; present-focused attention; recording and later evaluation; exploratory practice | “Now that I am more solid in my technique, these kinds of vocal issues almost never cause performance anxiety.” “recording his sessions and rather critiquing his practice afterwards.” | Supportive feedback and adaptive strategies restored control; external validation, criticism, and perfectionism intensified MPA. | “This doubt directly impacted on their performances through a greater intensity of MPA.” “negative perfectionism intensified their MPA.” |
| S16 | Music Self-Efficacy for Performance: An Explanatory Model Based on Social Support | Music students | Quantitative descriptive / SEM | Family support; teacher support; peer support | “family support... directly and mediated through musical performance anxiety.” “teachers and peers... relevant only for boys...” | None reported | — | Family support enhanced public-performance self-efficacy directly and through MPA; teacher/peer support was relevant mainly for boys. | “family support for self-efficacy in public performance: directly and mediated through musical performance anxiety.” |
| S17 | Norwegian music students’ perceptions and experiences of challenges and resources for health | Music students | Qualitative | Peers; teachers; audience synergy; rehearsal-room resources | “Social resources involved... social support from peers and teachers and synergy created between themselves and the audience through sharing of music.” “Environmental resources were linked to access to good rehearsal rooms.” | Situational understanding; adequate coping; high motivation; physical activity | “Personal resources included situational understanding, using adequate coping strategies, high motivation and participating in regular physical activity.” | Social and personal resources promoted health and helped students manage performance pressure. | “findings revealed several resources that promoted the students’ health...” |
| S18 | Past social support influences pre-performance self-confidence and performance quality among student musicians | Music students | Quantitative descriptive | Parents; past music teachers; current music teachers | “social support from the past teacher positively predicted pre-performance self-confidence and performance quality.” “current teacher positively predicted only performance quality...” | None reported | — | Past teacher support predicted pre-performance self-confidence and performance quality; current teacher support predicted performance quality only. | “Social support received from teachers by middle adolescence may be crucial for enhancing pre-performance self-confidence...” |
| S19 | Psychological predictors of music performance anxiety among vocal students: a gender-based SEM analysis | Music students | Quantitative descriptive / SEM | Parents; educators/teachers; peers | “social support from parents, educators, and peers...” | None reported | — | Support influenced MPA indirectly through psychological resources; indirect effects were stronger among female vocal students. | “self-efficacy, self-worth, and intrinsic motivation significantly mediated the relationship between social support and MPA.” “female students showing stronger indirect effects...” |
| S20 | Self-Regulated Learning in Music Education: A Structural Model of Social Support and Performance-Related Challenges | Music students | Quantitative descriptive / SEM | Teacher support; family/friend support | “Teacher social support is also positively associated with self-regulation...” “Family support is negatively associated with... helplessness...” | Self-regulated learning; practice time | “final model explains 52% of the variance in self-regulated learning.” | Teacher support promoted SRL; family/friend support was linked to lower helplessness. | “Support from family and friends is negatively associated with helplessness, indicating a protective role.” |
| S21 | Social Support and Performance Anxiety of College Music Students | Music students | Quantitative descriptive | Family; friends; significant others | “music majors perceived significantly lower levels of social support from significant others...” | None reported | — | General perceived support functioned as a psychosocial buffer against anxiety frequency and impact. | “Students with greater perceived social support reported less frequent anxiety and lower levels of impact of anxiety on ability to perform.” |
| S22 | Social support in university music students’ coping with performance anxiety: people, strategies and performance situations | Music students | Qualitative | One-to-one teachers; peers; accompanists | “one-to-one tuition teachers provide multifunctional strategies embedded in pedagogical practices, while peers provide emotional support and peer learning, and accompanists act as critical friends.” | Simulated performance; mental/visual rehearsal; accepting mistakes; relaxation; diet; absorbing activities; physical activity; virtual tools | “Among the eight MPA coping strategies, contrived performance situation... mental and visual rehearsal, accepting mistakes, relaxation skills, and dietary (teacher); absorbing activities and physical activities (peers); and virtual tools (accompanists).” | Social support improved performance skills and MPA coping skills. | “With social support, most participants have reported an improvement in their overall performance ability, including both performance skills and MPA coping skills.” |
| S23 | Student Musicians’ Personality Styles, Stresses, and Coping Patterns | Music students | Quantitative descriptive | Friends; informal support; formal counselling; specialized counsellors | “Friends were most often the support network. Formal counselling was seen as a last resort.” | Help-seeking; informal support; formal counselling | “Ninety-six per cent of the students sought help with their music-related stresses...” | Informal support was accessible, while formal counselling was a last resort, indicating barriers to professional help. | “Formal counselling was seen as a last resort.” |
| S24 | The role of coping strategy and experience in predicting music performance anxiety | Advanced students and professional musicians | Quantitative descriptive | Social-support seeking as coping dimension | “coping strategies based on demand of social support and avoidance strategies have a positive correlation with MPA.” | Social-support seeking; avoidance | “variance in MPA can be explained in part by experience... hours of individual practice per week and coping strategies based on social support and avoidance strategies.” | Seeking social support and avoidance co-occurred with higher MPA, suggesting compensatory or non-adaptive patterns. | “social support and avoidance strategies have a positive correlation with MPA.” |
| S25 | The role of music performance anxiety in musical training: four personal histories | Musicians/music trainees | Qualitative | Family support; ensemble/group experience; empathic teaching | “positive elements included family support, ensemble playing...” “Families played a key role in their children’s musical initiation...” | Visualization; cognitive analysis; self-medication; psychological techniques; nutrition/fluid control; beta-blockers | “To deal with symptoms of anxiety, several approaches were pursued: visualization, cognitive analysis, and self-medication.” | Family and ensemble experiences reduced anxiety; visualization and cognitive strategies were more adaptive, while self-medication was risky. | “family as the main reducing factor...” “Another way to reduce anxiety is by playing in an ensemble...” |
| S26 | Were you rushing or were you dragging? Perceived support, music-specific stress, and internalizing symptoms in university-level music students | Music students | Quantitative descriptive / moderation | Ensemble director support; private teacher support; music-major peer support | “social support from ensemble directors moderated the relationship between music-specific stress and depression.” | None reported | — | Ensemble director support protected high-stress students from stronger depression symptoms; no support source moderated stress–anxiety links. | “ensemble director support significantly moderated the relationship between music-specific stress and depression.” “no sources of support moderated the link between music-specific stress and anxiety.” |

**Table 2. MMAT Quality Assessment and Evidence Contribution Ratings for Included Studies**

| No. | Study | MMAT category | RQ1 evidence strength | RQ2 evidence strength | Q1 | Q2 | Q3 | Q4 | Q5 | Overall judgment |
| --- | --- | --- | --- | --- | --- | --- | --- | --- | --- | --- |
| S1 | An explanatory sequential pilot inquiry on music therapy and performance anxiety in university music education majors | Mixed methods | Strong | Moderate-to-strong | Yes | Yes | Yes | Yes | Yes | High |
| S2 | Associations between social support and help-seeking intention regarding music performance anxiety among Chinese music students: mediation via resilience and self-compassion | Quantitative descriptive / SEM | Strong | Strong | Can't tell | Yes | Yes | Can't tell | Yes | Moderate-to-high |
| S3 | Awareness and incidence of health problems among conservatoire students | Quantitative descriptive | Moderate | Moderate | Yes | Can't tell | Yes | Can't tell | Yes | Moderate-to-high |
| S4 | Clinical Trial of a Music Generated Vibrotactile Therapeutic Environment for Musicians: Main Effects and Outcome Differences Between Therapy Subgroups | Randomized controlled trial | Moderate-to-strong | Moderate | Yes | Yes | Can't tell | Can't tell | Yes | Moderate-to-high |
| S5 | Conservatory Students’ Music Performance Anxiety and Educational Expectations: A Qualitative Study | Qualitative | Moderate | Moderate | Yes | Yes | Yes | Yes | Yes | High |
| S6 | Effects of social support on music performance anxiety among university music students: chain mediation of emotional intelligence and self-efficacy | Quantitative descriptive / SEM | Strong | Strong | Can't tell | Yes | Yes | Can't tell | Yes | Moderate-to-high |
| S7 | Emotional Competence Within the Stress Coping Strategies of Music Education Students | Qualitative | Moderate | Moderate | Yes | Yes | Yes | Yes | Yes | High |
| S8 | Higher music education students’ experiences and management of performance anxiety: A qualitative study | Qualitative | Strong | Strong | Yes | Yes | Yes | Yes | Yes | High |
| S9 | How music education students embrace pre-performance rituals to enhance their craft | Mixed methods | Moderate | Moderate | Yes | Yes | Yes | Yes | Can't tell | High |
| S10 | “I feel like a fish out of water”: interpreting the occupational stress and well-being experiences of professional classical musicians | Qualitative | Moderate | Moderate | Yes | Yes | Yes | Yes | Yes | High |
| S11 | Intensive opera training program effects: A psychological investigation | Quantitative non-randomized | Moderate-to-strong | Moderate-to-strong | Can't tell | Yes | Yes | No | Yes | Moderate |
| S12 | ‘It made me feel helpless because I couldn’t control anything’: Occupational stress and well-being experiences of conservatoire music students | Qualitative | Moderate-to-strong | Moderate | Yes | Yes | Yes | Yes | Yes | High |
| S13 | “It’s Your Problem. Deal with It.” Performers’ Experiences of Psychological Challenges in Music | Qualitative | Strong | Moderate-to-strong | Yes | Yes | Yes | Yes | Yes | High |
| S14 | Music performance anxiety in Portuguese higher education: Contextual factors, perceptions, and strategies | Qualitative | Strong | Strong | Yes | Yes | Yes | Yes | Yes | High |
| S15 | Music Performance Anxiety, Perfectionism and Its Manifestation in the Lived Experiences of Singer-Teachers | Qualitative | Low-to-moderate | Low-to-moderate | Yes | Yes | Yes | Yes | Can't tell | Moderate-to-high |
| S16 | Music Self-Efficacy for Performance: An Explanatory Model Based on Social Support | Quantitative descriptive / SEM | Strong | Strong | Yes | Yes | Yes | Can't tell | Yes | High |
| S17 | Norwegian music students’ perceptions and experiences of challenges and resources for health | Qualitative | Moderate-to-strong | Moderate | Yes | Yes | Yes | Yes | Yes | High |
| S18 | Past social support influences pre-performance self-confidence and performance quality among student musicians | Quantitative descriptive | Strong | Strong | Can't tell | Yes | Yes | Can't tell | Yes | Moderate-to-high |
| S19 | Psychological predictors of music performance anxiety among vocal students: a gender-based SEM analysis | Quantitative descriptive / SEM | Strong | Strong | Can't tell | Yes | Yes | Can't tell | Yes | Moderate-to-high |
| S20 | Self-Regulated Learning in Music Education: A Structural Model of Social Support and Performance-Related Challenges | Quantitative descriptive / SEM | Strong | Strong | Can't tell | Yes | Yes | Can't tell | Yes | Moderate-to-high |
| S21 | Social Support and Performance Anxiety of College Music Students | Quantitative descriptive | Strong | Strong | Can't tell | Yes | Yes | Can't tell | Yes | Moderate-to-high |
| S22 | Social support in university music students’ coping with performance anxiety: people, strategies and performance situations | Qualitative | Strong | Strong | Yes | Yes | Yes | Yes | Yes | High |
| S23 | Student Musicians’ Personality Styles, Stresses, and Coping Patterns | Quantitative descriptive | Strong | Moderate | Yes | Can't tell | Yes | No | Yes | Moderate |
| S24 | The role of coping strategy and experience in predicting music performance anxiety | Quantitative descriptive | Strong | Strong | Yes | Yes | Yes | Can't tell | Yes | High |
| S25 | The role of music performance anxiety in musical training: four personal histories | Qualitative | Strong | Moderate | Yes | Yes | Yes | Yes | Can't tell | Moderate-to-high |
| S26 | Were you rushing or were you dragging? Perceived support, music-specific stress, and internalizing symptoms in university-level music students | Quantitative descriptive / moderation | Moderate-to-strong | Strong | Can't tell | Yes | Yes | Can't tell | Yes | Moderate-to-high |
